# Time- and NADPH-Dependent Inhibition on CYP3A by Gomisin A and the Pharmacokinetic Interactions between Gomisin A and Cyclophosphamide in Rats

**DOI:** 10.3390/molecules22081298

**Published:** 2017-08-08

**Authors:** Jianxiu Zhai, Feng Zhang, Shouhong Gao, Li Chen, Ge Feng, Jun Yin, Wansheng Chen

**Affiliations:** 1 School of Traditional Chinese Materia, Shenyang Pharmaceutical University, Shenyang 110016, China; lookingformilk@163.com; 2 Department of Pharmacy, Changzheng Hospital, Second Military Medical University, Shanghai 200433, China; fengzhang@smmu.edu.cn (F.Z.); gaoshouhong@126.com (S.G.); chenliky@163.com (L.C.); Gefeng1988@163.com (G.F.); 3 Key Laboratory of Jiangxi Province for Research on Active Ingredients in Natural Medicines, Bioengineering Research Institute, Yichun University, Yichun 336000, China

**Keywords:** cyclophosphamide, drug-drug interaction, gomisin A, pharmacokinetics, time-dependent inhibition

## Abstract

The traditional Chinese medicine *Schisandra chinensis* has remarkable protective effects against chemical-induced toxicity. Cyclophosphamide (CTX), in spite advances in chemotherapy and immunosuppressive regimes, is prone to cause severe toxicity due to its chloroacetaldehyde (CAA) metabolite produced by CYP3A. Our previous study identified that *S. chinensis* extract (SCE) co-administration potently decreased CAA production and attenuated liver, kidney and brain injuries in CTX-treated rats. Gomisin A (Gom A) is proved to be one of the most abundant bioactive lignans in *S. chinensis* with a significant CYP3A inhibitory effect. To find out whether and how Gom A participated in the chemoprevention of SCE against CTX toxicity, the Gom A-caused CYP3A inhibition in vitro as well as the pharmacokinetic interactions between Gom A and CTX in vivo were examined in this study. Using human liver microsomes, a reversible inhibition assay revealed that Gom A was a competitive inhibitor with a *K*_I_ value of 1.10 µM, and the time- and NADPH-dependent CYP3A inhibition of Gom A was observed in a time-dependent inhibition assay (*K*_I_ = 0.35 µM, *k*_inact_ = 1.96 min^−1^). Hepatic CYP3A mRNA expression experienced a significant increase in our rat model with Gom A administration. This explained why CAA production decreased in the 0.5 h- and 6 h-pretreatment rat groups while it increased in the 24 h- and 72 h-pretreatment groups, indicating a bidirectional effect of Gom A on CYP3A-mediated CTX metabolism. The present study suggested that Gom A participates like SCE in the pharmacokinetic intervention of CTX by blocking CYP3A-mediated metabolism and reducing CAA production, and thus plays an important role in the chemopreventive activity of *S. chinensis* against CTX toxicity, in addition to the previously recognized protective effects. Also, the combined use of *S. chinensis* preparation or other drugs containing Gom A as the main component with CTX needed to be addressed for better clinical intervention.

## 1. Introduction

Although chemotherapy has been an integral part of cancer treatment for decades, the numerous side effects are a major concern limiting its usage and causing patients’ unwanted clinical consequences in the long-term. As natural products, traditional Chinese medicines have been shown to be an excellent and reliable resource for cancer chemoprevention [1]. According to recent investigations, the traditional Chinese medicine *Schisandra chinensis* (*S. chinensis*) has been demonstrated to possess remarkable protective effects against chemical-induced toxicity [2,3] in addition to its widely known beneficial effects on the liver, kidney and nervous system in both experiment animals as well as in vitro human cell incubations [4,5,6,7]. Therefore, in our previous study the combined therapeutic regime of *S. chinensis* extract (SCE) with CTX exhibited desirable ameliorating effects on CTX toxicity, with decreased levels of some biochemical indexes such as serum marker enzymes. Additionally, a significant change in CTX pharmacokinetic parameters was observed along with the toxicity attenuation [8]. It was then hypothesised and preliminarily proven that the attenuation of toxicity could be at least partially attributed to CYP3A inhibition by SCE as well as a direct protective effect of SCE on tissues, as SCE has been reported to inhibit CYP3A activity in vivo [9].

**Scheme 1:**
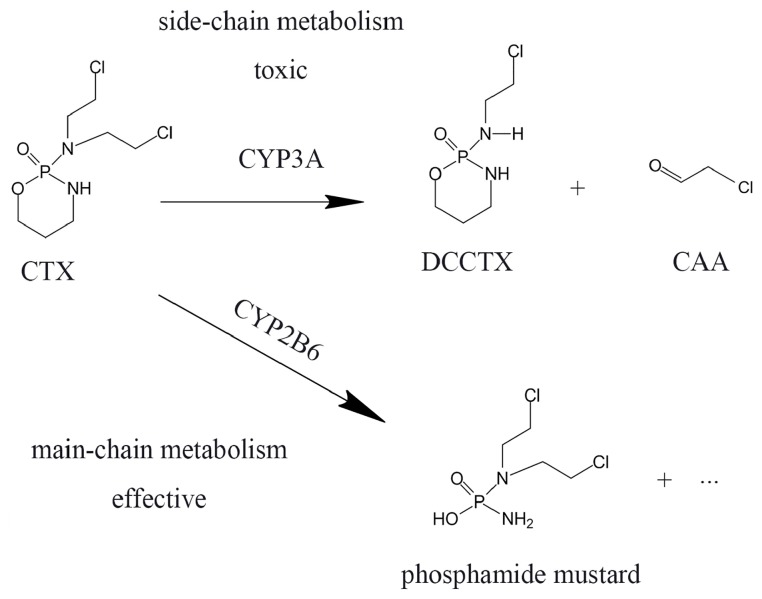
Metabolic pathways of CTX.

CTX is an alkylating anticancer drug widely employed in chemotherapy and immunosuppressive therapy [10]. It is mainly activated by CYP2B6 and then metabolized into the effective component phosphoramide mustard [11] (Scheme 1).

**Figure 1:**
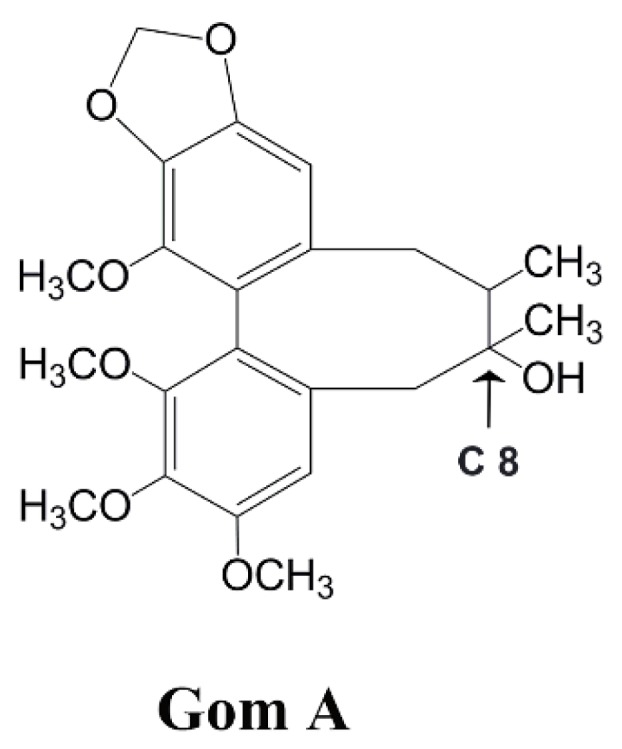
Chemical structure of Gom A.

Apart from that, a portion of CTX is metabolized by CYP3A into equimolar amounts of an inactive metabolite, 2-dechloroethylcyclophosphamide (DCCTX) and chloroacetaldehyde (CAA) as a by-product [11]. CAA was reckoned as the toxic product that might result in hepatotoxicity, neurotoxicity and nephrotoxicity [12,13]. In our previous study, a large decrease in the blood concentration of DCCTX and CAA was observed in CTX-treated rats with SCE co-administration [8]. Gomisin A (Gom A, Figure 1) is one of the most abundant bioactive lignans in *S. chinensis* [14].

As reported by Iwata and Wan et al., Gom A showed significant CYP3A inhibitory effect in vitro when co-incubated with human/rat liver microsomes (RLMs) and HepG2 cells [15,16]. However, the mechanism of CYP3A inhibition by Gom A, or the potential role of Gom A in the DDIs between SCE and CTX along with its detoxification effect of CTX through CYP3A inhibition are poorly understood.

So far, there is no report about the effect of Gom A on CTX metabolism and toxicity. Therefore this study aimed primarily to find out whether and how Gom A participates in the chemopreventive activity of *S. chinensis* against CTX toxicity, which was tested in in vitro incubation systems by using human liver microsomes (HLMs). Thereafter, the effects of Gom A on the toxic CYP3A-mediated CTX metabolism in rats was discussed based on the pharmacokinetic behaviors of DCCTX in rats with and without Gom A pretreatment.

## 2. Results

### 2.1. In Vitro CYP3A Inhibition Study

**Figure 2:**
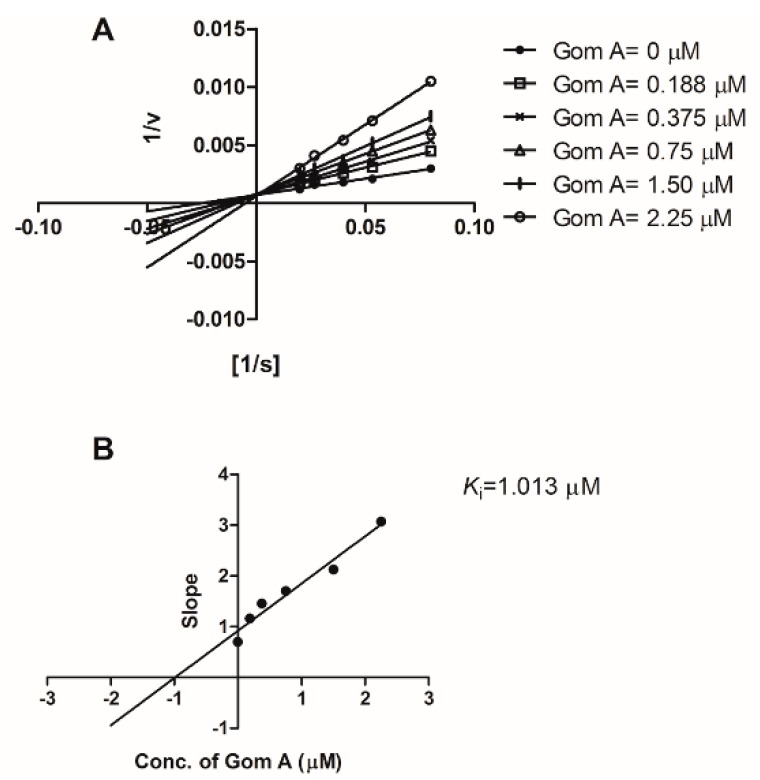
Lineweaver-Burk plot of Gom A on human liver CYP3A activity (**A**) and secondary plot of the slope taken from Lineweaver-Burk plot versus Gom A concentration (**B**). HLMs (0.5 mg protein/mL), different concentrations of Tes (12.5, 18.75, 25, 37.5 μM) and Gom A (0.375, 0.75, 1.5, 3 μM) were used. Each data point represents the mean of triplicate determinations.

The inhibitory effect of Gom A on CYP3A was investigated using a testosterone (Tes) 6 β-hydroxylation test with HLMs. By analyzing the Lineweaver-Burk plot of the enzyme kinetic data (Figure 2A), Gom A exhibited the characteristics of a competitive inhibitor with *K*_I_ value of 1.01 µM (Figure 2B).

**Figure 3:**
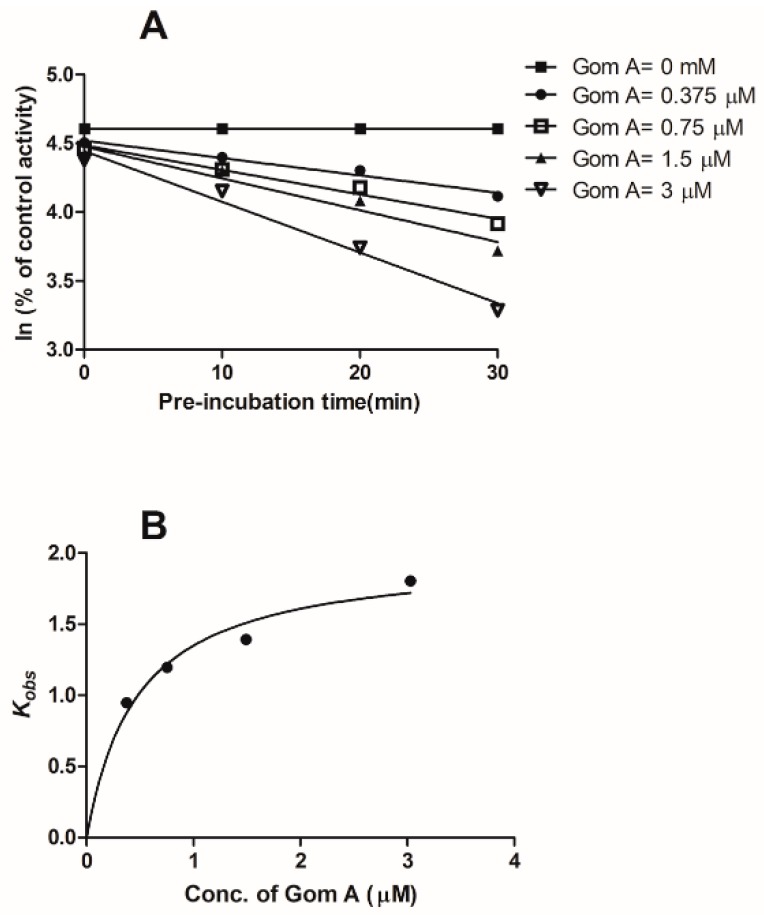
Inactivation of CYP3A4-catalyzed testosterone 6b-hydroxylase in human liver microsomes by Gom A. HLMs (0.5 mg protein/mL) were preincubated with 1% acetonitrile (vehicle control) or different concentrations of Gom A (0.375, 0.75, 1.5 and 3 μM) and NADPH-gs at 37 °C for 0, 10, 20 and 30 min in 100 mM PBS. (**A**) The plot of the percentage of control activity versus preincubation time; (**B**) The plot of the observed rates (*k*_obs_) versus different concentrations of Gom A, which was used to calculate the kinetic parameters *K*_I_ and *k*_inact_. Each data point represents the mean of triplicate determinations.

When preincubated with NADPH and HLMs for 30 min before combining with Tes, IC_50_ value shifted lower from 0.76 µM (without preincubation) to 0.42 µM. The time-dependent effect (1.8-fold shift in IC_50_) met the criteria of a 1.5-fold shift to indicate TDI [17]. By an additional NADPH-dependence assay, the CYP3A-inhibition was found more potent when Gom A was preincubated with NADPH (data not shown). The *k*_obs_ values were determined using linear regression analysis of the time course data at various concentrations of Gom A (Figure 3A). The *K*_I_ of 0.35 µM and the *k*_inact_ of 1.96 min^−1^ were obtained (Figure 3B), resulting in an inactivation efficiency (*k*_inact_/*K*_I_) of 5.64 mL min^−1^ mol^−1^.

**Figure 4:**
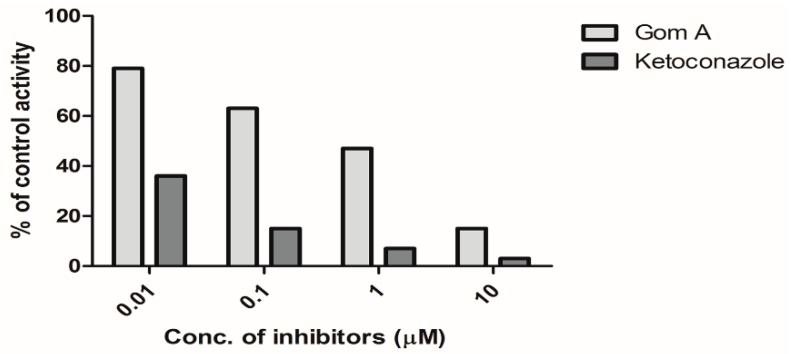
Inhibition of DCCTX production in HLMs by Gom A and Keto (0.01, 0.1, 1, and 10 μM) when incubated with CTX (100 ng/mL). HLMs (0.5 mg protein/mL) were preincubated with Gom A for 5 min in 100 mM PBS, the reaction was started by addition of NADPH-gs to the system. The mixture was incubated for 5 min at 37 °C. Each point represents the mean of triplicate determinations.

As CYP3A-mediated Tes 6 β-hydroxylation was strongly inhibited by Gom A in a concentration-dependent manner, the effect of Gom A on the production of DCCTX was also studied using HLMs. It was found out that 10 µM of Gom A potently decreased DCCTX production (15% of that in control group, Figure 4).

### 2.2. The Effect of Gom A Pretreatment on Rat Hepatic CYP3A Activity

**Figure 5:**
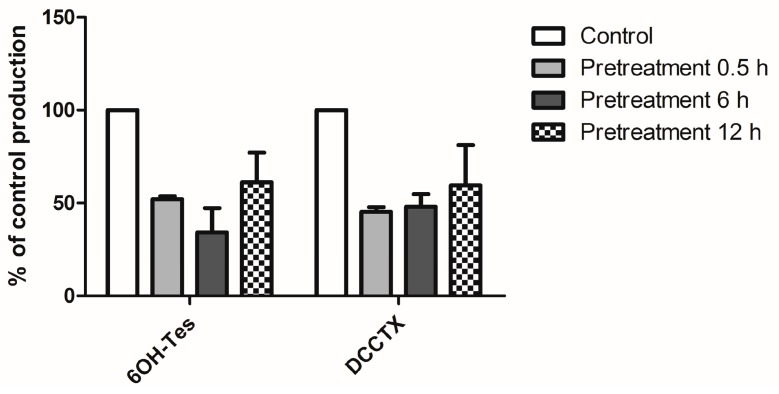
Effect of Gom A pretreatment on CYP3A activity in rat liver microsomes. Gom A was pretreated at single-dose of 20.8 mg/kg (50 µmol/kg). The rat livers were collected 0.5 h, 6 h and 12 h after Gom A administration. Production of 6OH-Tes and DCCTX were determined. Each point represents the mean of triplicate determinations.

As shown in Figure 5, the rat hepatic CYP3A activity was significantly decreased when pretreated with Gom A. At 0.5 h, 6 h and 12 h after Gom A administration, the rat hepatic CYP3A activities were 52.1%, 37.3% and 61.2% of the control group activity, respectively. The DCCTX production decreased by 54.7%, 52.0% and 40.5% compared with that of control group, respectively.

### 2.3. CYP3A Expression Assay

**Figure 6:**
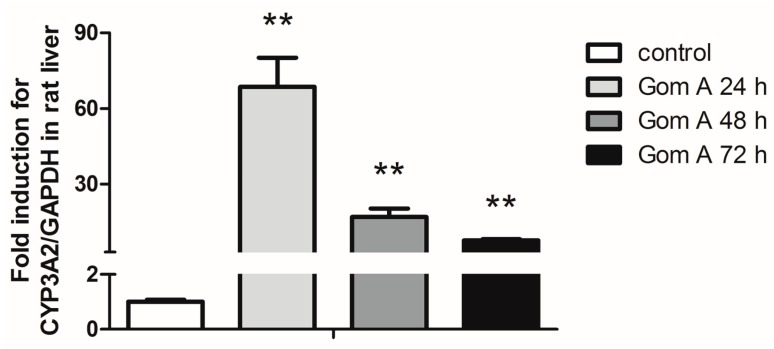
Effect of Gom A on rat liver CYP3A mRNA expression. Gom A was administrated at single-dose of 20.8 mg/kg (50 µmol/kg). The hepatic CYP3A2 mRNA expression was determined 24, 48 and 72 h after Gom A administration and was normalized to GAPDH. Results were mean ± SD of five rats for each group.

The rat hepatic CYP3A mRNA expression from groups A−D was determined 24, 48 and 72 h after single-dose administration of Gom A. Twenty-four hours after Gom A administration, the hepatic CYP3A mRNA expression was markedly increased by 73-fold compared with the vehicle group. Significant increased hepatic CYP3A mRNA expression was also observed 48 and 72 h after Gom A administration (18- and 8-fold, Figure 6).

### 2.4. In Vivo Pharmacokinetic Study

**Figure 7:**
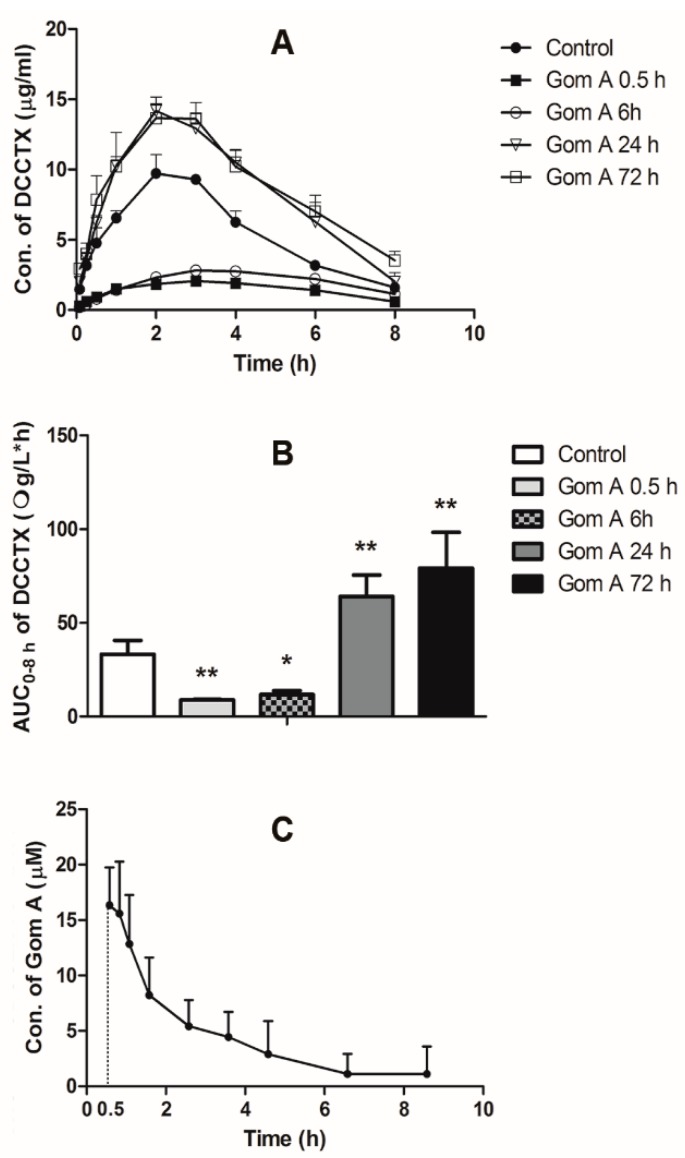
Blood concentration profiles of (**A**) DCCTX; (**B**) AUC_0–8h_ of DCCTX; (**C**) Gom A after CTX administration in group 1−5; (**D**) in group 1−5. Group 1, CTX alone group, 10 mL/kg saline (i.g.), Group 2−5 were given Gom A (i.g.) at the dose of 20.8 mg/kg (50 μmol/kg). In group 1, rats were administrated with CTX 0.5 h after saline administration. In group 2−5, rats were administrated with CTX (i.v.) 0.5 h, 6 h, 24 h and 72 h after Gom A pretreatment, respectively.

The effect of Gom A on CYP3A metabolism of CTX was further investigated in rats. DCCTX was assayed to reflect CAA production using a UHPLC-MS method (II). The pharmacokinetic profiles of DCCTX were studied when Gom A was administered 0.5, 6, 24 and 72 h (groups 2, 3, 4 and 5 respectively) prior to CTX administration. The blood concentration versus time curves of DCCTX and Gom A were shown in Figure 7, and the pharmacokinetic parameters are shown in Table 1.

When Gom A was administered 0.5 h and 6 h before CTX injection, the DCCTX production was significantly reduced and the C_max_ values of DCCTX in groups 2 and 3 were decreased markedly from 8.6 ± 1.3 µg/mL (group 1, CTX alone group) to 1.9 ± 0.2 µg/mL (group 2, 0.5 h, *p* < 0.01) and 2.6 ± 0.5 µg/mL (group 3, 6 h, *p* < 0.01) respectively. The AUC_0–8h_ values of DCCTX in groups 2 and 3 were decreased to 8.9 ± 0.3 µg·h/mL and 11.8 ± 2.5 µg·h/mL, which were 24% (*p* < 0.01) and 36% (*p* < 0.05) of group 1 (CTX alone group) (Figure 7B).

No significant change in t_1/2_ was observed in groups 2 and 3. However, Gom A showed an inductive effect on DCCTX production when pretreated to rats 24 h and 72 h before CTX injection. The C_max_ values of DCCTX were increased to 16.2 ± 1.2 µg/mL (group 4, 24 h, 1.9-fold, *p* < 0.01) and 20.0 ± 3.1 µg/mL (group 5, 72 h, 2.3-fold, *p* < 0.01). In groups 4 and 5, the AUC_0–8h_ values were markedly increased by 2.0- and 2.4-fold (*p* < 0.01) (Figure 7B).

The pharmacokinetic behavior of Gom A was also investigated using blood samples collected from group 2. As shown in Figure 7C, the observed maximal blood concentration of Gom A was approximately 16 µM in vivo.

## 3. Discussion

Previous research about the combination treatment of CTX and traditional Chinese medicines (TCMs) often focused on the changes of pharmacological and/or biochemical indexes, ignoring the potential occurrence of DDIs. This study confirmed that Gom A played an important role in the DDIs between *S. chinensis* and CTX, and thus might contribute to the observed chemoprotective effects of *S. chinensis* against CTX toxicity. The above results could lead to the conclusion that Gom A pretreatment might efficiently reduce the production of the toxic CTX metabolite CAA by inactivating CYP3A to some extent. To be exact, Gom A exhibited a multifaceted effect on CYP3A-mediated CTX metabolism with different time intervals between Gom A pretreatment and CTX administration.

Wan et al. and Iwata et al. pointed out that Gom A could cause moderate to strong CYP3A inhibition when co-incubated with HLMs or human HepG2 hepatoma cells [15,16]. Results from our study confirmed and extended previous findings, and characterized for the first time the time- and NADPH-dependent CYP3A inhibition of Gom A. In detail, Figure 2A (Lineweaver-Burk plot) suggests that Gom A was a competitive inhibitor in reversible inhibition assay, as little change of *V*_m_ was observed. In CYP3A TDI assessment, the IC_50_ value of Gom A decreased by 1.8-fold with a preincubation step, meeting the 1.5-fold shift criteria [17]. Since TDI had been closely related to mechanism-based inactivation (MBI) [18], in vitro data suggested that Gom A was likely to cause a long-term inhibition of CYP3A activity in rats, as the inactivated P450 enzyme had to be replaced to restore activity [19].

In HLMs, Gom A significantly inhibited CYP3A-mediated CTX metabolism. A better understanding was obtained that Gom A exhibited multifaceted effects on DCCTX production in vivo from subsequent pharmacokinetic data, which could be explained by the short- and long-term effect of Gom A administration on CYP3A-mediated metabolism of CTX. Therefore, Gom A and CTX were co-administered to rats with different time intervals. When Gom A was pretreated 0.5 h and 6 h before CTX injection, the production of DCCTX was significantly decreased. However, an increase in DCCTX blood concentration was observed when Gom A was pretreated 24 h and 72 h before CTX administration (Figure 7A, Table 1).

When CTX was administered within 6 h after Gom A pretreatment, the blood exposure of DCCTX was strongly decreased (Figure 7A, Table 1). As shown in Figure 7C, the blood concentration of Gom A reached a peak at or before the first blood sample collection and then decreased rapidly in experimental rats. Therefore, CTX was administered to rats with a much lower exposure of Gom A in group 3 (6 h time interval) compared with group 2 (0.5 h time interval). However, the pharmacokinetic parameters of DCCTX in groups 2 and 3 were similar. Moreover, according to Figure 5, the hepatic CYP3A in rat did not regain activity within 12 h after Gom A administration. The above observations indicated CYP3A inactivation, which agreed well with the in vitro study. Nevertheless, Qin et al. reported that Gom A co-administration had little effect on the in vivo metabolism of intravenously administered tacrolimus, which was also metabolized by CYP3A [20]. One possible explanation for the different outcomes could be a difference in plasma protein binding rates of the two chemicals. Tacrolimus is strongly bound to plasma protein, whereas the plasma protein binding rate of CTX is only approximately 9% [21,22]. With relatively low hepatic extraction ratios, only unbounded tacrolimus and CTX could be eliminated by hepatic CYPs [23,24,25]. With a much lower protein binding rate, it is possible that the hepatic CYP3A metabolism of CTX is more prone to be affected by CYP3A inhibitors compared with that of tacrolimus.

When CTX was administered 24 h and 72 h after Gom A pretreatment, a substantial increase in DCCTX production was observed. The major contributor to the increase could be CYP3A induction, as Gom A had been found as a potent inducer of CYP3A [26]. Figure 6 shows that the hepatic CYP3A mRNA expression in rats was substantially increased after single-dose Gom A pretreatment. The obtained data suggests that the CYP3A inductive effect by Gom A in rats lasted for more than 3 days, as the mRNA expression was still increased at 72 h after Gom A pretreatment compared with the control group. In general, the effect of Gom A on DCCTX production was a composite of CYP3A inhibition and induction. It is possible that though CYP3A mRNA expression kept accumulating within 24 h after Gom A administration, the CYP3A induction effect was covered up by CYP3A inactivation. 24 h after Gom A pretreatment, though a portion of CYP3A was still inactivated, the CYP3A inactivation was not of sufficient magnitude to have an effect in vivo with the substantially augmented hepatic CYP3A content.

In our previous study, the time interval between *S. chinensis* and CTX administration was 0.5 h, which meant than Gom A exhibited a strong inhibitory effect on rat hepatic CYP3A activity when CTX was injected. CAA is the CTX metabolite produced by CYP3A, which could result in broad toxic effects on the liver, kidney and nervous system [12,27,28,29]. It was demonstrated that Gom A acted as an important component in *S. chinensis* during the detoxification of CTX by inhibiting CYP3A activity and consequently reducing CAA blood concentrations. Furthermore, it was possible that this detoxification effect was not limited to the pharmacokinetic interference by Gom A. CAA could cause GSH depletion and lipid peroxidation, ultimately resulting in cytotoxicity and cell death [30,31]. Gom A treatment has been reported to greatly increase the total liver and mitochondrial GSH levels in mice [32]. Thus, Gom A may enhance the resistance against CTX toxicity by increasing GSH levels in experiment rats. As the major bioactive component of *S. chinensis*, Gom A has also been indicated to attenuate chemical-induced damages in liver and kidney by modulating NRF2/ARE and MAPK signal pathways [4,6]. In summary, it could be concluded that Gom A was an essential component in *S. chinensis* for the chemoprevention against CTX toxicity.

The current study was aimed primarily to evaluate the in vivo effects of Gom A pretreatment on CYP3A-mediated CTX metabolism and investigate the pharmacokinetic behaviors of Gom A in rats. Gom A contains the methylenedioxyphenyl group in common with another *Schisandrae* lignan gomisin C, which had been found to potently inactivate CYP3A [15]. Methylenedioxyphenyl-containing compounds could be transformed by CYPs into a reactive carbene metabolite, and the carbene metabolite is apt to react with CYPs to form a catalytically inactive metabolic-inhibitor (MI) complex, which had been demonstrated to play an important role in CYPs inactivation [19,33].

Several limitations in this study need to be addressed. Our in vitro study only provided indirect evidence for the Gom A-induced MBI on CYP3A. Future investigations need to be performed to investigate the specific mechanisms of the CYP3A inactivation induced by Gom A. In addition, only HLMs was used in CYP3A inhibition investigations. In further mechanism studies, both rat and human liver microsomes should be involved to provide a more comprehensive mechanism description. Also, to provide visual evidence of the protective effects of Gom A against CTX toxicity, changes in related biochemical indexes of CTX-treated rats with and without Gom A pretreatment should be further investigated.

## 4. Materials and Methods

### 4.1. Chemicals and Reagents

DCCTX standard (purity > 98%) was obtained from Toronto Research Chemicals Inc. (Toronto, ON, Canada). CTX (batch No. 5H071A) and Mesna injections (Endoxan, batch No. 4A177A) were obtained from Baxter International Inc. (Deerfield, MA, USA). Male human liver microsomes (HLMs) (mixed, lot No. M0202A), testosterone (Tes), 6β-hydroxytestosterone (6OH-Tes), phenacetin (IS-I of 6OH-Tes), tinidazole (TNZ, IS-II of DCCTX), bifendate (IS-III of Gom A), NADP+, glucose-phosphate dehydrogenase, potassium phosphate buffer (PBS, 0.1 M, pH 7.4), ketoconazole (Keto) and magnesium chloride hexahydrate were obtained from Meilun Biotechnology Co., Ltd. (Dalian, China). Glucose-6-phosphate was obtained from Sigma Aldrich (St. Louis, MO, USA). Gom A standard was isolated and purified from *S. chinensis*, which purity was determined by high-performance liquid chromatography (HPLC) with UV detection (>98%, Supplementary Figure S1). Acetonitrile and methanol of HPLC grade were obtained from Merck (Darmstadt, Germany). HPLC grade of formic acid was obtained from Tedia (Fairfield, OH, USA). All other reagents were of analytical grade. BCA protein assay kit was obtained from Nanjing Jiancheng Bioengineering Institute (Nanjing, China). RNeasy Mini Kit was obtained from Qiagen (Hilden, Germany). RNase Zap spray, DEPC treated water and SYBR Select Master Mix was obtained from Invitrogen (Carlsbad, CA, USA).

### 4.2. Animals

Seventy male Sprague-Dawley rats (200−220 g) were obtained from Shanghai Slac Laboratory Animal Co., Ltd. (Shanghai, China). All the rats were acclimated for at least one week under controlled room temperature (22−24 °C) and humidity (55−60%) with a day/night cycle (12-h light and 12-h dark). The rats were fasted for 12 h before the experiments. All experiments were performed according to the guidelines of Experimental Animal Administration issued by the Ministry of Science and Technology of the People’s Republic of China (http://www.most.gov.cn) and were approved by the Laboratory Animal Ethics Committee of the Second Military Medical University (SMMU, License No. 20160310, 20170720).

### 4.3. In Vitro CYP3A Inhibition Study

A NADPH regenerating system (NADPH-gs, containing 10 mM glucose-6-phosphate, 5 mM magnesium chloride, 1 U/mL glucose-6-phosphate dehydrogenase and 1 mM NADP+) was constructed. The metabolite of Tes, 6OH-Tes, was assayed by UHPLC-MS method (I) for CYP3A activity assessment.

#### 4.3.1. CYP3A Inhibition Assays

The CYP3A inhibition by Gom A was preliminarily evaluated. In a total volume of 200 µL, several concentrations of Gom A (0.08, 0.31, 0.63, 2.5, 10 and 40 µM) were incubated with HLMs in PBS (0.1 M) containing 25 µM Tes (near *K*_m_, Supplementary Figure S2), 0.5 mg/mL HLMs and NADPH-gs at 37 °C. After a 5 min warm up period, reactions were initiated with addition of NADPH-gs. Incubations were conducted at 37 °C for 10 min and then terminated by the addition of 200 µL ice-cold acetonitrile containing 18 ng/mL phenacetin (IS-I). To determine the *K*_i_ value, Gom A (0.38, 0.75, 1.5, 3 µM) was added to the reaction mixture containing different concentrations of Tes (12.5, 18.75, 25, 37.5 and 50 µM) in the incubation systems. Incubations were processed and terminated as described earlier.

#### 4.3.2. IC_50_ Shift Assays

To identify time-dependent inhibition (TDI), the IC_50_ values of Gom A were determined using HLMs with and without a 30 minute preincubation step. In a final volume of 100 µL, Gom A at different concentrations (0, 0.08, 0.31, 0.63, 2.5, 10 and 40 µM) was preincubated with 10 × HLMs (5 mg/mL) at 37 °C with NADPH-gs in PBS (0.1 M). After 30 min, the incubation mixture was diluted 10 folds using PBS (0.1 M). Then 100 µL of the diluted mixture was added into a secondary incubation containing NADPH-gs and 25 µM Tes. The final volume was 200 µL. After 10 min incubation, the reaction was terminated by the addition of 200 µL ice-cold acetonitrile containing 18 ng/mL phenacetin (IS-I).

To confirm the NADPH dependence of the CYP3A inhibition by Gom A, additional preincubation experiments were conducted. In brief, Gom A (1 µM) was preincubated with 10 × HLMs (5 mg/mL) at 37 °C for 30 min with and without NADPH-gs, the preincubation mixture was then diluted, followed by the steps described above.

#### 4.3.3. *K*_I_ and *k*_inact_ Assays

To determine *K*_I_ and *k*_inact_ values, 0, 0.38, 0.75, 1.5 and 3 µM of Gom A was preincubated with 10 × HLMs at 37 °C with NADPH-gs in PBS for 0, 10, 20 and 30 min. The following steps were similar as described in the IC_50_ shift assays. The concentration of Tes was set as 100 µM (well beyond *K*_m_) to avoid reversible inhibition. All the incubations were performed in triplicate.

#### 4.3.4. Effect of Gom A and Ketoconazole on DCCTX Production in HLMs

The effect of Gom A on CYP3A metabolism of CTX in HLMs was investigated. CTX was metabolized by CYP3A into two metabolites DCCTX (inactive) and CAA (toxic) in equimolar amount [11]. Considering the difficulty for direct CAA determination, DCCTX was assayed to reflect CAA production using an established UHPLC-MS method (II).

The CYP3A inhibitor ketoconazole (Keto) was used as positive control. Briefly, various concentrations of Gom A and Keto (0.01, 0.1, 1 and 10 µM) were incubated with HLMs (0.5 mg/mL) in PBS (0.1 M) containing NADPH-gs and 100 µM CTX in a total volume of 200 µL. After a 5 min warm up period, reactions were initiated with addition of NADPH-gs. Incubations were conducted at 37 °C for 60 min and then terminated by the addition of 200 µL ice-cold acetonitrile containing 6 ng/mL of TNZ (IS-II).

### 4.4. The Effect of Gom A Pretreatment on Rat Hepatic CYP3A Activity

Rats were randomly divided into four groups (control group; pretreatment groups: 0.5 h group, 6 h group, 12 h group). Rats in control group and pretreatment groups were administrated (i.g.) with saline (2 mL/kg) and Gom A (20.8 mg/kg) respectively. In control group, rats were anesthetized 0.5 h after administration. In pretreatment groups, rats were anesthetized 0.5 h, 6 h, 12 h after Gom A pretreatment respectively. After anesthesia, the rat livers were infused with cool saline. After infusion, rats were euthanized and livers were collected. The liver microsomes were prepared using calcium precipitation method [34]. Briefly, livers were weighted, washed with cool PBS (0.1 M), and then homogenized in cool Tris-PBS (50 mM Tris, *w*/*v* = 1:4). The mixture was centrifuged at 14,000 *g* for 20 min at 4 °C. The supernatant was mixed with CaCl_2_ solution (88 mM, *v*_supernantant_/*v*_CaCl2_ = 10:1) and then centrifuged at 14,000 *g* for 20 min at 4 °C. The precipitate was resuspended in Tris-PBS and centrifuged (14,000 *g*, 4 °C, 20 min). Finally, the precipitate was resuspended in PBS containing 250 mM glucose, giving a concentration of 5 mg protein/mL. The prepared microsomes were stored at −80 °C until use.

To determine the effect of Gom A pretreatment on rat hepatic CYP3A activity, liver microsomes from control and pretreatment groups were incubated with Tes (25 µM) and CTX (100 ng/mL) respectively. The incubation procedures were similar with Section 4.3.1 and Section 4.3.4 for Tes and CTX incubation, respectively. For incubations using Tes, 0.5 mg/mL RLMs was incubated with 25 µM Tes in PBS (0.1 M) containing NADPH-gs at 37 °C in a total volume of 200 µL. After a 5 min warm up period, reactions were initiated with addition of NADPH-gs. Incubations were conducted at 37 °C for 10 min and then terminated by the addition of 200 µL ice-cold acetonitrile containing 18 ng/mL phenacetin (IS-I). For incubations using CTX, 0.5 mg/mL RLMs was incubated with 100 ng/mL CTX in PBS (0.1 M) containing NADPH-gs at 37 °C in a total volume of 200 µL. After a 5 min warm up period, reactions were initiated with addition of NADPH-gs. Incubations were conducted at 37 °C for 60 min and then terminated by the addition of 200 µL ice-cold acetonitrile containing 6 ng/mL of TNZ (IS-II).

After reaction termination, concentration of 6OH-Tes and DCCTX were determined.

### 4.5. CYP3A Expression Assay

#### 4.5.1. Drug Administration

Rats were randomly divided into four groups (groups A−D, *n* = 5), respectively. In brief, rats in group A (vehicle control group) were i.g. administered with saline (10 mL/kg), and were euthanized 72 h later. Rats in groups B−D were i.g. administered with Gom A at dose of 20.8 mg/kg (50 µmol/kg, same with pharmacokinetic study), and euthanized 24, 48 and 72 h respectively after Gom A treatment. After euthanization, rat livers were immediately collected, washed with ice-cold saline, and stored at −80 °C for CYP3A protein expression assay.

#### 4.5.2. Quantitative Reverse Transcriptase PCR (Real-Time PCR)

To determine CYP3A mRNA content in rat liver, the liver samples were homogenized and the total RNA was extracted from liver samples using RNeasy Mini Kit according to the manufacturer’s instructions (Qiagen). The concentration of total RNA was determined by UV spectra at 260/280 nm, and the purity was determined by calculating the ration of UV absorbance. 2 µg of total RNA in a total volume of 50 µL (1 µg/25 µL) was reverse transcribed into template cDNA using dNTP mix (1 mM), random primers (0.5 µg/mL), AMV RT (22 U/µL), MgCl_2_ (25 mM) and RT buffer (10×).

For qRT-PCR, forward and reverse primer sequences (Table 2) of CYP3A gene were used. In a Stratagene Mx3005P sequence detection system (Agilent Technologies Agilent, Santa Clara, CA, USA), 50 ng of cDNA/sample and 10 µM forward and reverse primers were added to a total reaction mixture of 20 µL of SYBR Select Master Mix (Invitrogen). GAPDH was applied as the house-keeping gene. All assays were performed in triplicate. After 2 min at 50 °C and 10 min at 95 °C, amplifications were achieved with 40 repeating cycles at 95 °C for 15 s and 60 °C for 1 min, followed by a dissociation stage at 95 °C for 1 min, 55 °C for 30 s and 95 °C for 30 s. The CYP3A mRNA levels of groups A−D were expressed as a ratio of induced to vehicle control group (group A).

### 4.6. In Vivo Pharmacokinetic Study

#### 4.6.1. Drug Administration

Rats were randomly divided into five groups (groups 1−5, *n* = 6), respectively. To determine the short- and long-term effect of single-dose Gom A pretreatment on CYP3A metabolism of CTX, Gom A and CTX were administered to rats with different time intervals in groups 2−5. In brief, rats in group 1 (CTX alone group) were i.g. administered with saline (10 mL/kg, vehicle control), and rats in groups 2−5 were given Gom A (i.g.) at the dose of 20.8 mg/kg (50 μmol/kg). Rats in group 1 were intravenously (i.v.) administered with CTX (300 mg/kg) 0.5 h after saline administration. In groups 2–5, rats were given CTX (i.v.) 0.5, 6, 24 and 72 h after Gom A pretreatment, respectively. Mesna was injected (420 mg/kg, i.v.) 1 hour after CTX administration in all groups to prevent bladder injury in accordance with clinical practice.

#### 4.6.2. Sample Collection

Blood samples were collected from the postocular vein at 0.083, 0.25, 0.5, 1, 2, 3, 4, 6, 8, 12 and 24 h after CTX injection. Samples were transferred to heparin tubes and centrifuged at 5000 rpm for 15 min. Plasma was obtained and stored at −20 °C until required for further analysis.

### 4.7. UHPLC-MS/MS Analysis

Assays of 6OH-Tes, CTX, and DCCTX were performed on an Agilent 1290 series UHPLC system (Agilent Technologies, Waldbronn, Germany) coupled to an Agilent 6460 triple-quadrupole mass spectrometer system (Agilent Technologies, Wilmington, DE, USA). Assay of Gom A was performed on an Agilent 1200 series system coupled to an Agilent 6410 triple-quadrupole mass spectrometer system (Agilent Inc., Lexington, MA, USA). All samples were determined under a positive electrospray ionization (ESI) mode with the spray voltage set at 4000 V. System was set in the multiple reaction monitoring (MRM) mode.

UHPLC-MS/MS method (I): 6OH-Tes concentrations were analyzed according to the previous reports with slight modifications [36] (Supplementary Figure S3). The terminated sample were centrifuged at 14,000 rpm for 10 min, 10 μL of each supernatant was injected into the UHPLC-MS/MS system for analysis. In general, an Agilent Elipse Plus 2.1 × 50 mm column packed with 1.8 µm C18 was applied. The column temperature was set as 30 °C at a flow rate of 0.3 mL/min. The mobile phase was consisted of aqueous 1% formic acid with the addition of 10 mM ammonium acetate solution (A) and methanol (B) with the gradient elution program set as follow: 5–50–80% B from 0–1–4 min. The ion transitions were *m*/*z* 305.0→269.0 for 6OH-Tes and *m*/*z* 181.0→111.0 for phenacetin (IS-I). UHPLC-MS method (II): DCCTX concentrations were determined using our previously developed UHPLC-MS/MS method [37] (Supplementary Figure S4). For pharmacokinetic study, plasma samples were diluted 30-fold with plasma from drug-free rats. After vortexing for 2 min, 100 μL of each diluted sample was mixed with 400 μL acetonitrile (containing 6 ng/mL TNZ (IS-II), vortexed, and centrifuged at 14,000 rpm for 10 min. 100 μL supernatant of each sample was mixed with 400 μL aqueous solution containing 10% acetonitrile followed by a rough centrifugation. For HLMs inhibition study, samples were vortexed for 1 min after reaction termination, and the mixture was then centrifuged at 14,000 rpm for 10 min. After centrifugation, the obtained supernatant (5 μL) was injected into the UHPLC-MS/MS system, using methanol/10 mM ammonium acetate aqueous solution as the mobile phase at a flow rate of 0.25 mL/min. The ion transitions were set as *m*/*z* 199.2→78 for DCCTX and *m*/*z* 248.1→121.1 for TNZ (IS-II). UHPLC-MS/MS method (III): Gom A concentrations were determined using slightly modified LC/MS method which developed by our laboratory [38]. Briefly, a 100 µL plasma sample was transferred to a 10 mL centrifuge tube with addition of 20 µL IS solution and 20 µL water/acetonitrile (90:10, *v*/*v*), vortexed for 30 s and then extracted using 3 mL methyl tertiary butyl ether. After extraction, the sample was centrifuged at 5000 rpm for 10 min. The organic layer was quantitatively transferred to a 5 mL glass tube and evaporated to dryness at 35 °C. Then the dried extract was reconstituted in 100 µL solvent (water–acetonitrile, 20:80, *v*/*v*) followed by injection of 10 µL aliquot into LC-MS/MS system. Separation was performed on a Zorbax SB-C18 reserved-phase column (100 mm × 2.1 mm i.d., 3.5 µm) with the mobile phase consisting of acetonitrile, methanol and water −0.1% formic acid (72:18:10, *v*/*v*/*v*) at a flow rate of 0.3 mL/min. The ion transitions were set as *m*/*z* 399→329 for Gom A and *m*/*z* 387.0→145.0 for bifendate (IS-III).

According to methodological validation (see Supplementary Information: Methodological Validation of 6OH-Tes, DCCTX and Gom A), the linearity was good when the concentration of 6OH-Tes was in the range of 15.4−985.5 nM, the concentration of DCCTX was in the range of 5−1000 μg/mL and the concentration of Gom A was in the range of 4.7−300 ng/mL, respectively. The extraction recovery was 72.9% for DCCTX and 74.3% for Gom A in plasma. The inter- and intra-day precision was less than 11.5% for 6OH-Tes, less than 9.4% for DCCTX and less than 11.0% for Gom A, respectively. The inter-and intra-batch accuracy was less than 6.7% for 6OH-Tes, less than 9.7% for DCCTX and less than 11.7% for Gom A, respectively.

### 4.8. Data Analysis

Pharmacokinetic profiles of CTX and DCCTX were estimated by one-compartmental model using the DAS 2.0 software (Data Access Service, Medical College of Wannan, Anhui, China). Data were expressed as mean ± SD. Comparisons between two groups were performed using one-way ANOVA followed by LSD test in the condition of variance homogeneity or Dunnett T3 test in the case of variance heterogeneity. The differences were considered to be statistically significant at * *p* < 0.05 and ** *p* < 0.01. IC_50_ values of Gom A were determined by nonlinear regression analysis using GraphPad Prism 5.01 software (GraphPad Software Inc., San Diego, CA, USA). The mode of inhibition was verified by Lineweaver-Burk plots of the enzyme kinetic data, and the *K*_I_ value was determined by linear regression analysis with GraphPad Prism 5.01 (GraphPad Software Inc.). The initial inactivation rate constant (*k*_obs_, min^−1^) was estimated from the initial slope of the linear regression line at each concentration of Gom A. Then the values of *k*_obs_ were plotted versus Gom A concentration, and the maximal inactivation rate constant *k*_inact_, along with the concentration of inactivator (at which the rate of the inactivation is half maiximal) *K*_I_ values, were determined using Equation (1). I is the inhibitor concentration in the primary incubation:

*k*_obs_ = (*k*_inact_*I)/(*K*_I_ + I) (1)

## 5. Conclusions

In conclusion, this study suggested that Gom A, in addition to its previous recognized protective effects, plays an important role in the chemoprevention of *S. chinensis* against CTX toxicity by blocking CYP3A-mediated metabolism and reducing CAA production. Also, the combined use of *S. chinensis* preparation or other drugs containing Gom A as the main component with CTX needs to be addressed for better clinical intervention.

## Figures and Tables

**Table 1:** Pharmacokinetic parameters of DCCTX after CTX (300 mg/kg, i.v.) administration, with different time intervals after Gom A administration, in groups 1−5 *^a^*.

DCCTX in Group 1−5 *^a^*	C_max_	T_max_	t_1/2_	MRT_0–8h_	AUC_0–8h_
	µg/mL	h	h	h	µg *h/mL
Group 1	8.6 ± 1.3	2.0 ± 0.1	2.2 ± 0.5	3.3 ± 0.2	32.5 ± 8.4
Group 2	1.9 ± 0.2 **	2.9 ± 0.6 *	3.1 ± 0.9	3.8 ± 0.2 **	8.9 ± 0.3 **
Group 3	2.6 ± 0.5 **	3.7 ± 0.8 **	3.2 ± 0.8	4.2 ± 0.1 **	11.8 ± 2.5 *
Group 4	16.2 ± 1.2 **	2.0 ± 0.1	2.0 ± 0.4	3.3 ± 0.2	65.9 ± 12.4 **
Group 5	20.0 ± 3.1 **	3.3 ± 0.5 **	3.8 ± 1.2 *	3.7 ± 0.2 **	79.2 ± 19.1 **

*^a^* Group 1: control group, 10 mL/kg saline; groups 2−5: Gom A at doses of 20.8 mg/kg (50 µmol/kg) was co-administrated. Rats in group 1 were intravenously administered with CTX (300 mg/kg) 0.5 h after saline administration. Rats in group 2−5 were intravenously administered with CTX 0.5, 6 h, 24 h and 72 h after Gom A administration, respectively. Data are the mean ± S.D. (*n* = 6). One-way analysis of variance with post hoc test was conducted. * *p* < 0.05 from control group; ** *p* < 0.01 from control group.

**Table 2:** List of Primer Sequences Used for RT-PCR.

Gene	Forward Primer (5′−3′)	Reverse Primer (5′−3′)	Reference
CYP3A2	GAA TGC TTT TCTGTC TTC ACA AAC C	TTT ACC AAA ATG TCT CCA TAC TGT TCA	[35]
GAPDH	CCC ATC ACC ATC TTC CAG GAG	GTT GTC ATG GAT GAC CTT GGC	

## Supplementary Materials

Supplementary Materials are available online.

## Abbreviations

ANOVA	analysis of variance
AUC	area under concentration-time curve
C_max_	peak plasma concentration
CAA	chloroacetaldehyde
CL	clearance
CTX	cyclophosphamide
DCCTX	2-dechloroethylcyclophosphamide
CYP450	cytochrome P450
DDIs	drug-drug interactions
Gom A	gomisin A
Gom C	gomisin C
HLMs	human liver microsomes
IC_50_	half maximal inhibitory concentration
IS	internal standard
Keto	ketoconazole
K_i_	dissociation constant for reversible inhibition
*k*_obs_	initial inactivation rate constant
*k*_inact_	the maximal inactivation rate constant
K_I_	half maximal inhibitory concentration for MBI
LLOQ	lower limit of quantification
LSD	least significant difference
MBI	mechanism-based inhibition
MI	metabolite-intermediate
MRT	mean residence time
NADPH	dihydronicotinamide adenine dinucleotide phosphate
NADPH-gs	NADPH regenerating system
PBS	potassium phosphate buffer
SCE	*Schisandra chinensis* extract
SD	standard deviation
t_1/2_	terminal half-life
Tes	testosterone
TDI	time-dependent inhibition
T_max_	time of plasma concentration reach a maximum
TNZ	tinidazole
V_max_	maximum reaction rate
Vd	apparent volume of distribution
6OH-Tes	6β-hydroxytestosterone.

## References

[1] Wang S., Penchala S., Prabhu S., Wang J., Huang Y. (2010). Molecular basis of traditional Chinese medicine in cancer chemoprevention. Curr. Drug Discov. Technol..

[2] Ip S.-P., Che C.-T., Kong Y.-C., Ko K.-M. (2001). Effects of schisandrin B pretreatment on tumor necrosis factor-α induced apoptosis and Hsp70 expression in mouse liver. Cell Stress Chaperones.

[3] Kim S.R., Lee M.K., Koo K.A., Kim S.H., Sung S.H., Lee N.G., Markelonis G.J., Oh T.H., Yang J.H., Kim Y.C. (2004). Dibenzocyclooctadiene lignans from *Schisandra chinensis* protect primary cultures of rat cortical cells from glutamate-induced toxicity. J. Neurosci. Res..

[4] Hwang I.S., Kim J.E., Lee Y.J., Kwak M.H., Choi Y.H., Kang B.C., Hong J.T., Hwang D.Y. (2013). Protective effects of gomisin A isolated from *Schisandra chinensis* against CCl4-induced hepatic and renal injury. Int. J. Mol. Med..

[5] Jiang B., Li S., Liu W., Yang Y., Chen W., He D., Cheng X., Wang Z., Chen W., Wang C. (2015). Inhibitive activities detection of monoamine oxidases (MAO) A and B inhibitors in human liver MAO incubations by UPLC-ESI-MS/MS. J. Pharm. Biomed. Anal..

[6] Jiang Y., Fan X., Wang Y., Tan H., Chen P., Zeng H., Huang M., Bi H. (2015). Hepato-protective effects of six schisandra lignans on acetaminophen-induced liver injury are partially associated with the inhibition of CYP-mediated bioactivation. Chem.-Biol. Interact..

[7] Kim D.H., Hung T.M., Bae K.H., Jung J.W., Lee S., Yoon B.H., Cheong J.H., Ko K.H., Ryu J.H. (2006). Gomisin A improves scopolamine-induced memory impairment in mice. Eur. J. Pharmacol..

[8] Feng G., Zhai J., Chen W., Xiong X., Gao S., Xia T., Zhang F. (2017). The effects of *Schisandra sphenathera* and *Schisandra chinensis* extract on the metabolism of cyclophosphamide in rats. China J. Hosp. Pharm..

[9] Lai L., Hao H., Wang Q., Zheng C., Zhou F., Liu Y., Wang Y., Yu G., Kang A., Peng Y. (2009). Effects of short-term and long-term pretreatment of Schisandra lignans on regulating hepatic and intestinal CYP3A in rats. Drug Metab. Dispos. Biol. Fate Chem..

[10] Binotto G., Trentin L., Semenzato G. (2003). Ifosfamide and cyclophosphamide: Effects on immunosurveillance. Oncology.

[11] De Jonge M.E., Huitema A.D., Rodenhuis S., Beijnen J.H. (2005). Clinical pharmacokinetics of cyclophosphamide. Clin. Pharmacokinet..

[12] McDonald G.B., Slattery J.T., Bouvier M.E., Ren S., Batchelder A.L., Kalhorn T.F., Schoch H.G., Anasetti C., Gooley T. (2003). Cyclophosphamide metabolism, liver toxicity, and mortality following hematopoietic stem cell transplantation. Blood.

[13] Rzeski W., Pruskil S., Macke A., Felderhoff-Mueser U., Reiher A.K., Hoerster F., Jansma C., Jarosz B., Stefovska V., Bittigau P. (2004). Anticancer agents are potent neurotoxins and in vivo. Ann. Neurol..

[14] Lu Y., Chen D.F. (2009). Analysis of *Schisandra chinensis* and Schisandra sphenanthera. J. Chromatogr. A.

[15] Iwata H., Tezuka Y., Kadota S., Hiratsuka A., Watabe T. (2004). Identification and characterization of potent CYP3A4 inhibitors in Schisandra fruit extract. Drug Metab. Dispos..

[16] Wan C.K., Tse A.K., Yu Z.L., Zhu G.Y., Wang H., Fong D.W. (2010). Inhibition of cytochrome P450 3A4 activity by schisandrol A and gomisin A isolated from Fructus *Schisandrae chinensis*. Phytomedicine.

[17] Grimm S.W., Einolf H.J., Hall S.D., He K., Lim H.K., Ling K.H., Lu C., Nomeir A.A., Seibert E., Skordos K.W. (2009). The conduct of in vitro studies to address time-dependent inhibition of drug-metabolizing enzymes: A perspective of the pharmaceutical research and manufacturers of America. Drug Metab. Dispos..

[18] Atkinson A., Kenny J.R., Grime K. (2005). Automated assessment of time-dependent inhibition of human cytochrome P450 enzymes using liquid chromatography-tandem mass spectrometry analysis. Drug Metab. Dispos..

[19] Kalgutkar A.S., Obach R.S., Maurer T.S. (2007). Mechanism-based inactivation of cytochrome P450 enzymes: chemical mechanisms, structure-activity relationships and relationship to clinical drug-drug interactions and idiosyncratic adverse drug reactions. Curr. Drug Metab..

[20] Qin X.L., Chen X., Wang Y., Xue X.P., Wang Y., Li J.L., Wang X.D., Zhong G.P., Wang C.X., Yang H. (2014). In vivo to in vitro effects of six bioactive lignans of Wuzhi tablet (Schisandra sphenanthera extract) on the CYP3A/P-glycoprotein-mediated absorption and metabolism of tacrolimus. Drug Metab. Dispos..

[21] Medeiros M., Valverde S., Del Moral I., Velásquez-Jones L., Hernández A.M., Castañeda-Hernández G., Reyes H., Filler G. (2016). Are Tacrolimus Pharmacokinetics Affected by Nephrotic Stage?. Ther. Drug Monit..

[22] Wang L.H., Lee C.S., Majeske B.L., Marbury T.C. (1981). Clearance and recovery calculations in hemodialysis: Application to plasma, red blood cell, and dialysate measurements for cyclophosphamide. Clin. Pharmacol. Ther..

[23] Juma F., Rogers H., Trounce J. (1979). First pass hepatic metabolism of cyclophosphamide [proceedings]. Br. J. Clin. Pharmacol..

[24] Möller A., Iwasaki K., Kawamura A., Teramura Y., Shiraga T., Hata T., Schäfer A., Undre N. (1999). The disposition of 14C-labeled tacrolimus after intravenous and oral administration in healthy human subjects. Drug Metab. Dispos..

[25] Verbeeck R.K. (2008). Pharmacokinetics and dosage adjustment in patients with hepatic dysfunction. Eur. J. Clin. Pharmacol..

[26] Mu Y., Zhang J., Zhang S., Zhou H.H., Toma D., Ren S., Huang L., Yaramus M., Baum A., Venkataramanan R. (2006). Traditional Chinese medicines Wu Wei Zi (*Schisandra chinensis Baill*) and Gan Cao (*Glycyrrhiza uralensis Fisch*) activate pregnane X receptor and increase warfarin clearance in rats. J. Pharmacol. Exp. Ther..

[27] Dubourg L., Tanière P., Cochat P., Baverel G., Michoudet C. (2002). Toxicity of chloroacetaldehyde is similar in adult and pediatric kidney tubules. Pediatr. Nephrol..

[28] Dubourg L., Michoudet C., Cochat P., Baverel G. (2001). Human kidney tubules detoxify chloroacetaldehyde, a presumed nephrotoxic metabolite of ifosfamide. J. Am. Soc. Nephrol..

[29] Goren M., Wright R., Pratt C., Pell F. (1986). Dechloroethylation of ifosfamide and neurotoxicity. Lancet.

[30] MacAllister S.L., Martin-Brisac N., Lau V., Yang K., O’Brien P.J. (2013). Acrolein and chloroacetaldehyde: An examination of the cell and cell-free biomarkers of toxicity. Chem.-Biol. Interact..

[31] Singh M., Dang T.N., Arseneault M., Ramassamy C. (2010). Role of by-products of lipid oxidation in Alzheimer’s disease brain: A focus on acrolein. J. Alzheimer’s Dis..

[32] Jiang Y.M., Wang Y., Tan H.S., Yu T., Fan X.M., Chen P., Zeng H., Huang M., Bi H.C. (2016). Schisandrol B protects against acetaminophen-induced acute hepatotoxicity in mice via activation of the NRF2/ARE signaling pathway. Acta Pharmacol. Sin..

[33] Fukuto J.M., Kumagai Y., Cho A.K. (1991). Determination of the mechanism of demethylenation of (methylenedioxy) phenyl compounds by cytochrome P450 using deuterium isotope effects. J. Med. Chem..

[34] Aitio A., Vainio H. (1976). UDPglucuronosyltransferase and mixed function oxidase activity in microsomes prepared by differential centrifugation and calcium aggregation. Basic Clin. Pharmacol. Toxicol..

[35] Myers A.L., Hassan H.E., Lee I.J., Eddington N.D. (2010). Repeated administration of oxycodone modifies the gene expression of several drug metabolising enzymes in the hepatic tissue of male Sprague-Dawley rats, including glutathione S-transferase A-5 (rGSTA5) and CYP3A2. J. Pharm. Pharmacol..

[36] Tolonen A., Petsalo A., Turpeinen M., Uusitalo J., Pelkonen O. (2007). In vitro interaction cocktail assay for nine major cytochrome P450 enzymes with 13 probe reactions and a single LC/MSMS run: Analytical validation and testing with monoclonal anti-CYP antibodies. J. Mass Spectrom..

[37] Chen L., Xiong X., Gao S., Zhai J., Chen W., Liu Z., Huang H., Zhang F. (2016). UHPLC-MS/MS in simultaneous determination of cyclophosphamide and its metabolites in rat plasma. Acad. J. Second Mil. Med. Univ..

[38] Wei H., Miao H., Yun Y., Li J., Qian X., Wu R., Chen W. (2014). Validation of an LC–MS/MS method for quantitative analysis of the 5 bioactive components of Wuzhi capsule in human plasma samples. Ther. Drug Monit..

