# Supplementary material for: Time- and NADPH-Dependent Inhibition on CYP3A by Gomisin A and the Pharmacokinetic Interactions between Gomisin A and Cyclophosphamide in Rats
Source: Molecules. 2017 Aug 8;22(8):1298. doi: 10.3390/molecules22081298 (PMC6152024; doi:10.3390/molecules22081298)
Supplement: Supplementary file 1 [file molecules-22-01298-s001.pdf]

Electronic Supplementary Information

Time- and NADPH-dependent inhibition on CYP3A by Gomisin A and the pharmacokinetic interactions between Gomisin A and cyclophosphamide in rats

Jianxiu Zhai [a,b], Feng Zhang[b], Shouhong Gao [b], Li Chen[b,c], Ge Feng[b,c], Jun Yin[a,*] and Wansheng Chen [b,*]

[a] School of Traditional Chinese Material, Shenyang Pharmaceutical University, Shenyang, China

[b] Department of Pharmacy, Changzheng Hospital, Second Military Medical University, Shanghai, China

[c] Key Laboratory of Jiangxi Province for Research on Active Ingredients in Natural Medicines, Bioengineering Research Institute, Yichun University, Yichun, China

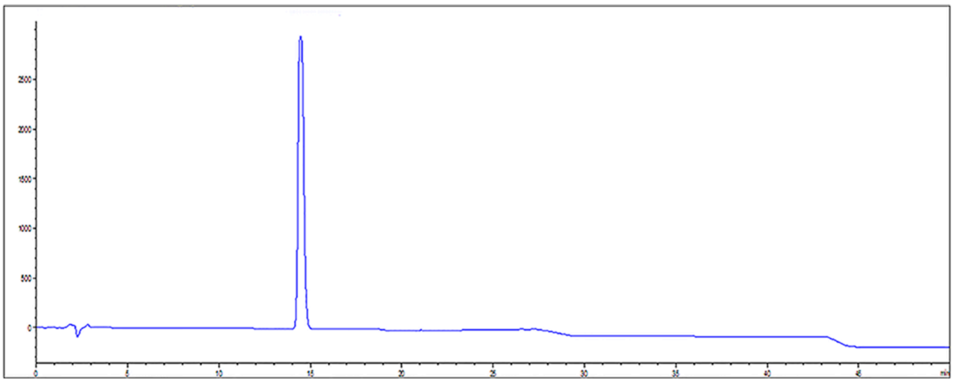

Supplement, Fig. 1. The HPLC figure of Gom A.

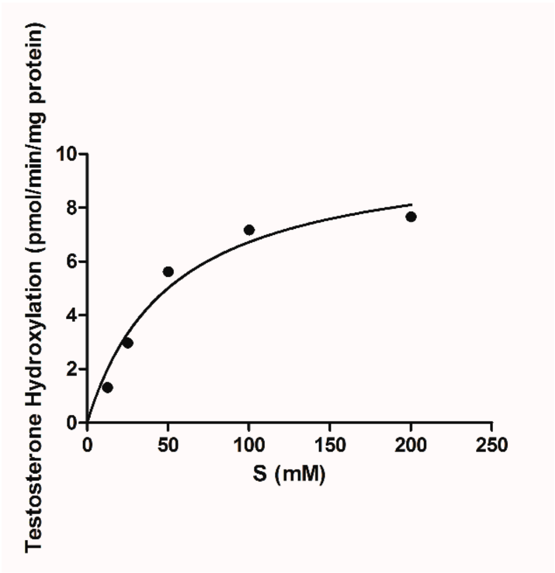

Supplement, Fig. 2. The metabolism kinetics of Tes in HLMs. Each point represents the mean of triplicate samples (n =3).

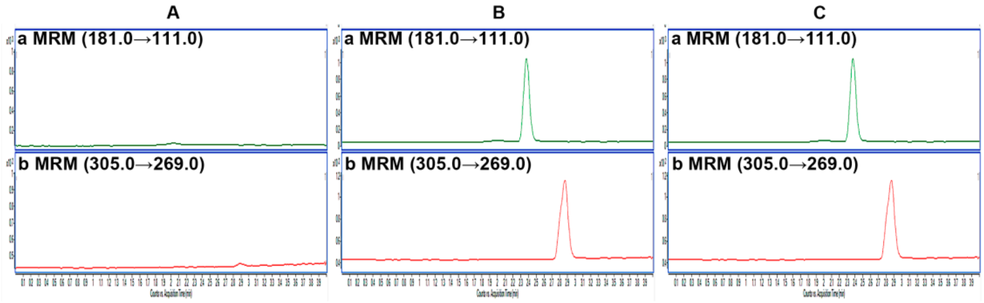

Supplement, Fig. 3. Representative MRM chromatograms of (a),phenacetin (IS-I); (b), 6OH-Tes; (A), A blank sample; (B), a blank sample spiked with 6OH-Tes at the lower limit of quantification and phenacetin (IS-I); (C), a sample after incubation.

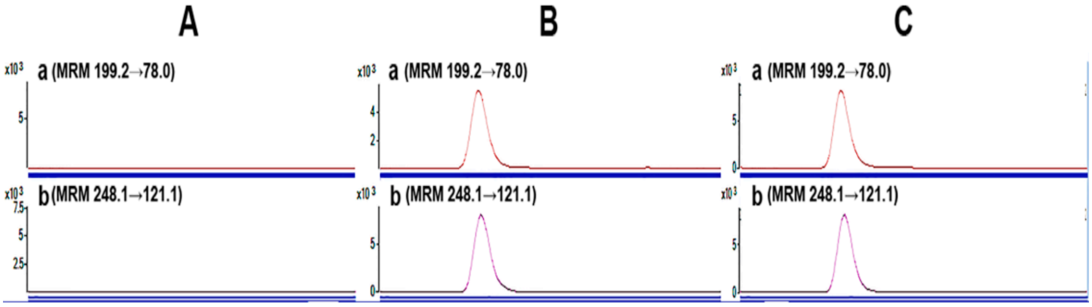

Supplement, Fig. 4. Representative MRM chromatograms of (a), DCCTX; (b), TNZ (IS-II) in rat plasma. (A), A blank plasma sample; (B), a blank plasma sample spiked with DCCTX at the lower limit of quantification and TNZ (IS-II); (C), a plasma sample from a rat 1 hour after administration of CTX.

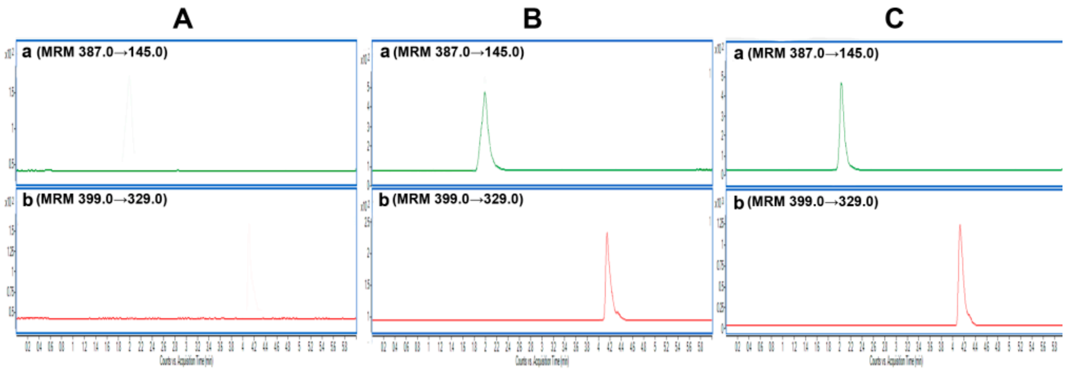

Supplement, Fig. 5. Representative MRM chromatograms of (a), bifendate (IS-III); (b), Gom A in rat plasma. (A), A blank plasma sample; (B), a blank plasma sample spiked with Gom A at the lower limit of quantification and bifendate (IS-III); (C), a plasma sample from a rat 5 min after administration of CTX and 35 min after administration of Gom A.

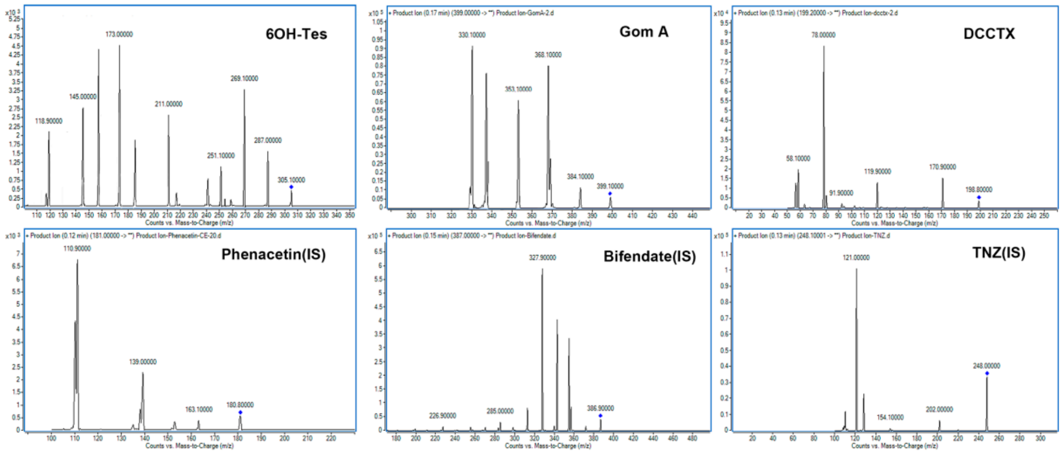

Supplement, Fig. 6. Full scan product ion of precursor ions of 6OH-Tes and phenacetin (IS-I of 6OH-Tes), DCCTX and TNZ (IS-II of DCCTX), Gom A and bifendate (IS-III of Gom A).

Supplementary file (1):

Methodological Validation of 6OH-Tes, DCCTX and Gom A

(1) 6OH-Tes

Linearity and sensitivity

The seven-point calibration curves were liner from 15.4 nM to 985.5 nM for 6OH-Tes with r^2 of 0.99 or better. The linearity was assessed by a weighted ($1/x^2$) least squares regression analysis from six intra- and inter-batch calibration curves. The back-calculated concentrations with percent relative error (%RE) and percent coefficient of variation (%CV) are shown in supplement Table 1. The LLOQ for paroxetine was found to be 15.4 nM. At LLOQ, the mean signal-to-noise ratios were obtained as 24.7.

Precision and accuracy

With each batch consisting of six replicates of QC samples at four concentration levels (LLOQQC, LQC, MQC, HQC), precision and accuracy for 6OH-Tes were summarized in supplement Table 2. The inter- and intra-batch precision were less than 11.5%. The inter-and intra-batch accuracy were between 1.4% and 6.7%.

Supplement Table 1. Linearity and sensitivity of back-calculated concentration of calibration samples for 6β-hydroxytestosterone (6OH-Tes)[a]

Analyte	Concentration added (nM)	Concentration found (nM) n=6	%CV	%RE
6OH-Tes	15.4	15.76	7.2	2.3
	30.8	30.54	11.6	-0.8
	61.6	57.23	15.9	-7.1
	123.2	136.77	7.6	11.0
	246.4	244.05	8.8	-0.9
	492.7	497.85	10.1	1.0
	985.5	912.47	8.9	-7.4

[a] %CV = percent coefficient of variation; %RE = percent relative error.

Supplement Table 2. Inter- and intra-batch precision and accuracy[a]

		Inter-batch (n=6)			Intra-batch (n=6)		
Analyte	Concentration added (nM)	%Recovery	%CV	%RE	%Recovery	%CV	%RE
6OH-Tes	15.4	106.7	5.9	6.7	105.9	7.9	5.9
	30.8	98.6	11.5	-1.4	94.7	11.1	-5.3
	492.7	102.2	5.3	2.2	103.4	10.2	3.4
	985.5	93.9	8.0	-6.1	94.4	10.5	-5.6

QC = quality control; %CV = percent coefficient of variation; HQC = high quality control; LQC = low quality control; MQC = medium quality control; %RE = percent relative error.

(2) DCCTX

Linearity and sensitivity

The eight-point calibration curves were liner from 5 μg/ml to 1000 μg/ml for DCCTX with r^2 of 0.99 or better. The linearity was assessed by a weighted ($1/x^2$) least squares regression analysis from six intra- and inter-batch calibration curves. The back-calculated concentrations with percent relative error (%RE) and percent coefficient of variation (%CV) were shown in supplement Table 3. The LLOQ for paroxetine was found to be 5 μg/ml. At LLOQ, the mean signal-to-noise ratios were obtained as 60.7.

Precision and accuracy

With each batch consisting of six replicates of QC samples at four concentration levels (LLOQQC, LQC, MQC, HQC), precision and accuracy for DCCTX were summarized in supplement Table 4. The inter- and intra-batch precision were less than 9.4%. The inter-and intra-batch accuracy were between 5.2% and 9.7%.

Extraction recovery

The extraction recovery of DCCTX from rat plasma was determined by six quality control samples at low, medium and high concentration. The mean recovery was 72.9% with %CV of 12.0%, as shown in supplement Table 5.

Supplement Table 3. Linearity and sensitivity of back-calculated concentration of calibration samples for DCCTX[a]

Analyte	Concentration added (μg/ml)	Concentration found (μg/ml) n=6	%CV	%RE
DCCTX	5.0	5.41	4.4	8.2
	10.0	11.08	5.9	10.8
	25.0	24.60	6.7	-1.6
	50.0	49.48	5.3	-1.0
	100.0	103.05	6.2	3.1
	250.0	279.72	7.5	11.9
	500.0	573.57	5.7	14.7
	1,000.0	1,086.83	7.8	8.7

[a] %CV = percent coefficient of variation; %RE = percent relative error.

Supplement Table 4. Inter- and intra-batch precision and accuracy[a]

		Inter-batch (n=6)			Intra-batch (n=6)		
Analyte	Concentration added (μg/ml)	%Recovery	%CV	%RE	%Recovery	%CV	%RE
DCCTX	5.0	107.8	9.4	7.8	109.7	7.3	9.7
	10.0	109.2	7.1	9.2	109.4	5.7	9.4
	500.0	107.4	2.8	7.4	105.2	5.2	5.2
	1000.0	105.2	6.8	5.2	106.1	6.2	6.1

QC = quality control; %CV = percent coefficient of variation; HQC = high quality control; LQC = low quality control; MQC = medium quality control; %RE = percent relative error.

Supplement Table 5. Extraction recovery for DCCTX (n=6) [a]

QC level	Response A	Response B	%Recovery	Mean Reconvery	%CV
10 ng/ml	14595.7	21106.8	69.9		
500 ng/ml	879357.0	1208256.4	73.7	72.9	12.0
1000 ng/ml	1654754.3	2237838.7	75.0		

[a]QC = quality control; %CV = percent coefficient of variation; LQC = low quality control; MQC= medium quality control; HQC = high quality control.

(3) Gom A

Linearity and sensitivity

The eight-point calibration curves were liner from 5 μg/ml to 1000 μg/ml for DCCTX with r^2 of 0.99 or better. The linearity was assessed by a weighted ($1/x^2$) least squares regression analysis from six intra- and inter-batch calibration curves. The back-calculated concentrations with percent relative error (%RE) and percent coefficient of variation (%CV) are shown in supplement Table 6. The LLOQ for paroxetine was found to be 4.7 ng/ml. At LLOQ, the mean signal-to-noise ratios were obtained as 21.6.

Precision and accuracy

With each batch consisting of six replicates of QC samples at four concentration levels (LLOQQC, LQC, MQC, HQC), precision and accuracy for DCCTX were summarized in supplement Table 7. The inter- and intra-batch precision were less than 11.0%. The inter-and intra-batch accuracy were between 0.2% and 11.7%.

Extraction recovery

The extraction recovery of Gom A from rat plasma was determined by six quality control samples at low, medium and high concentration. The mean recovery was 74.3% with %CV of 12.3%, as shown in supplement Table 8.

Supplement Table 6. Linearity and sensitivity of back-calculated concentration of calibration samples for Gom A[a]

Analyte	Concentration added (ng/ml)	Concentration found (μg/ml) n=6	%CV	%RE
Gom A	4.7	4.5	9.9	-4.3
	9.4	10.0	17.2	6.3
	18.8	20.0	11.4	6.9
	37.5	39.8	7.8	6.2
	75.0	83.0	7.1	10.6
	150.0	143.9	7.4	-4.1
	300.0	307.7	7.2	2.6

[a] %CV = percent coefficient of variation; %RE = percent relative error.

Supplement Table 7. Inter- and intra-batch precision and accuracy[a]

	Inter-batch (n=6)				Intra-batch (n=6)		
Analyte	Concentration added (ng/ml)	%Recovery	%CV	%RE	%Recovery	%CV	%RE
Gom A	4.7	97.2	10.9	-2.8	99.2	8.8	-0.8
	9.4	95.2	7.3	-1.7	104.6	11.0	7.5
	150.0	97.0	7.3	-3.0	111.7	9.5	11.7
	300.0	99.8	5.3	-0.2	103.5	8.2	3.5

QC = quality control; %CV = percent coefficient of variation; HQC = high quality control; LQC = low quality control; MQC = medium quality control; %RE = percent relative error.

Supplement Table 8. Extraction recovery for Gom A (n=6) [a]

QC level	Response A	Response B	%Recovery	Mean Reconvery	%CV
9.4 ng/ml	1161.8	1633.7	72.9	74.3	12.3
150 ng/ml	20390.8	26836.5	76.1		
300 ng/ml	34687.2	46990.2	73.9		

[a]QC = quality control; %CV = percent coefficient of variation; LQC = low quality control; MQC= medium quality control; HQC = high quality control.
